# Generation of synthetic antibody fragments with optimal complementarity determining region lengths for Notch-1 recognition

**DOI:** 10.3389/fmicb.2022.931307

**Published:** 2022-08-03

**Authors:** Bharathikumar Vellalore Maruthachalam, Kris Barreto, Daniel Hogan, Anthony Kusalik, Clarence Ronald Geyer

**Affiliations:** ^1^Department of Biochemistry, University of Saskatchewan, Saskatoon, SK, Canada; ^2^Department of Computer Science, University of Saskatchewan, Saskatoon, SK, Canada; ^3^Department of Pathology, University of Saskatchewan, Saskatoon, SK, Canada

**Keywords:** phage display, synthetic antibody, focused library, long CDR library, next-generation sequencing, Notch signaling, Notch1

## Abstract

Synthetic antibodies have been engineered against a wide variety of antigens with desirable biophysical, biochemical, and pharmacological properties. Here, we describe the generation and characterization of synthetic antigen-binding fragments (Fabs) against Notch-1. Three single-framework synthetic Fab libraries, named S, F, and modified-F, were screened against the recombinant human Notch-1 extracellular domain using phage display. These libraries were built on a modified trastuzumab framework, containing two or four diversified complementarity-determining regions (CDRs) and different CDR diversity designs. In total, 12 Notch-1 Fabs were generated with 10 different CDRH3 lengths. These Fabs possessed a high affinity for Notch-1 (sub-nM to mid-nM K_Dapp_ values) and exhibited different binding profiles (mono-, bi-or tri-specific) toward Notch/Jagged receptors. Importantly, we showed that screening focused diversity libraries, implementing next-generation sequencing approaches, and fine-tuning the CDR length diversity provided improved binding solutions for Notch-1 recognition. These findings have implications for antibody library design and antibody phage display.

## Introduction

Notch signaling is a highly conserved pathway that influences multiple cell fate decisions, including proliferation, apoptosis, differentiation, migration, and angiogenesis in developing and adult metazoan organisms. In mammals, Notch signaling is initiated by four Notch receptors (Notch1-4) and five Delta/Serrate/LAG-2 (DSL) ligands (Jagged-1/2 and DLL-1/3/4), all of which are modular, type-I, single-pass, transmembrane proteins. The extracellular region of Notch contains a series of epidermal growth factor (EGF)-like repeats that are required for ligand binding, followed by a negative regulatory region, and a hetero-dimerization domain. The extracellular region of DSL ligands contains an MNNL domain (module at the N-terminus of Notch ligands), followed by a DSL domain, and a series of EGF-like repeats similar to Notch receptors. The intracellular region of Notch contains numerous well-defined domains, whereas the intracellular region is not conserved in DSL ligands (reviewed by Katoh and Katoh, 2020).

Canonical, short-range Notch signaling happens between two adjacent cells, the signal-sending cell, which expresses the DSL ligand, and the signal-receiving cell, which expresses the Notch receptor. A bi-molecular interaction between the Notch receptor and the DSL ligand at the cell surface initiates a process called regulated intramembrane proteolysis where the Notch receptor is cleaved within the extracellular hetero-dimerization domain by a metalloprotease of the ADAM family. This DSL-dependent metalloprotease processing renders Notch sensitive to the γ-secretase protein complex. γ-Secretase cleaves the Notch receptor within the transmembrane region, releasing the Notch intracellular domain. The intracellular domain translocates into the nucleus where it forms a transcriptional activation complex and regulates Notch-responsive genes (reviewed by Katoh and Katoh, 2020).

In addition to its physiological roles, deregulation of Notch signaling is associated with developmental disorders, neurological diseases, solid cancers, and hematologic malignancies. These pathological conditions result from overexpression or gain/loss-of-function mutations within Notch and Jagged receptors (Ranganathan et al., 2011). Small molecules have been developed that block Notch signaling by inhibiting the γ-secretase complex and monoclonal antibodies have been developed that that target Notch or Jagged ectodomains (Zhou et al., 2022). Since γ-secretase inhibitors block the proteolysis of multiple transmembrane proteins, including all four Notch receptors, they cause side effects in clinical trials. Similar side effects are observed with pan-Notch-specific monoclonal antibodies (Takebe et al., 2014). Synthetic antibodies have been constructed that target the negative-regulatory region of Notch-1 and the receptor-binding region of Jagged-1 and bind specifically to Notch and Jagged receptors, respectively. They exhibit potent and selective inhibition of Notch-1 or Jagged-1 signaling in tumor models and show promising results in pre-clinical studies (Wu et al., 2010; Lafkas et al., 2015). Thus, there is interest in developing paralogue-specific, synthetic antibodies against Notch receptors and DSL ligands.

Previously, we designed, constructed, and validated a phage-displayed, single-framework, synthetic antigen-binding fragment (Fab) library named library-S (Maruthachalam et al., 2017). We used Library-S and a similar synthetic Fab library, named Library-F (Persson et al., 2013) for generating high-affinity Fabs against Notch-2/3 and Jagged-2 (Barreto et al., 2019). In this work, we describe the generation and characterization of Notch-1 Fabs from three synthetic Fab libraries S, F, and modified-F. These Fab libraries were built on a modified trastuzumab framework, containing two or four diversified complementarity determining regions (CDRs) and different CDR diversity designs. During the course of the Fab generation, we showed that screening focused diversity libraries, implementing next-generation sequencing (NGS) approaches, and fine-tuning the library diversity, can improve binding solutions for Notch-1 recognition. We generated 12 Notch-1 Fabs with high affinity (sub-nM to mid-nM K_Dapp_ values) and different binding specificities toward Notch/Jagged receptors. The highest-affinity Fab bound specifically to Notch-1 with a K_Dapp_ value of 0.16 ± 0.1 nM. Notch-1 Fabs described in this work should permit a more precise modulation of the Notch signaling pathway.

## Results

### Notch-1 Fabs from Library-S

**Figure 1:**
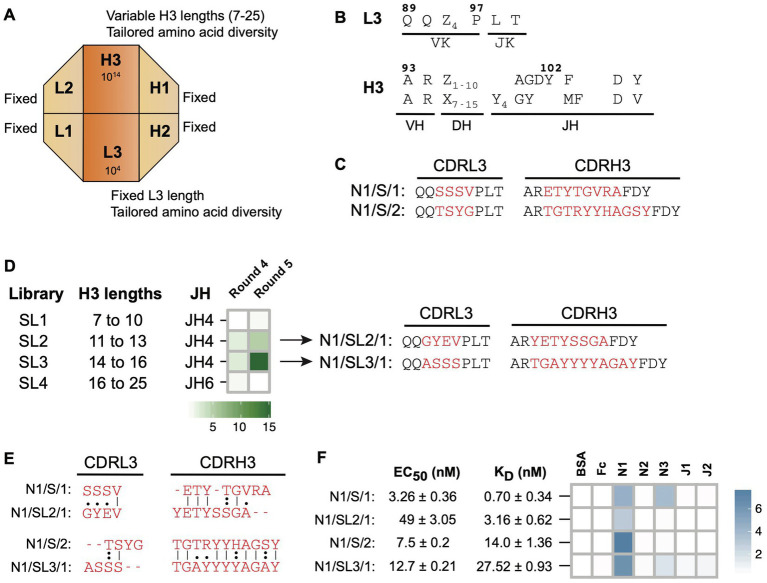
Notch-1 Fabs from Library-S. **(A)** Schematic representation of Library-S CDR diversity. **(B)** CDR diversity designs for L3 and H3. Z denotes any of the following 13 amino acids introduced at different proportions: Y (20%), S (20%), G (20%), T (6.5%), A (6.5%), P (6.5%), H (3.5%), R (3.5%), E (3.5%), F (2.5%), W (2.5%), V (2.5%), or L (2.5%). X denotes any of the following nine amino acids introduced at different proportions: Y (25%), S (20%), G (20%), A (10%), F (5%), W (5%), H (5%), P (5%), or V (5%). CDRH3 length is varied by altering the number of Z and X. **(C)** CDRL3 and CDRH3 sequences of two Notch-1 Fabs isolated from Library-S. Fixed anchor residues are in black and diversified CDR positions are in red. **(D)** Left panel: panning of Library-S sub-libraries (SL1–SL4) against Notch-1. Heatmap showing the enrichment of Notch-1 binding phages after phage display selection rounds 4 and 5. Fold enrichment is the ratio of number of phages eluted from target-coated wells to number of phages eluted from BSA-coated wells. Right panel: CDRL3 and CDRH3 sequences of Fab N1/SL2/1 (isolated from SL2) and Fab N1/SL3/1 (isolated from SL3). **(E)** Pairwise sequence alignments between master-and sub-library Fab CDR sequences. Only diversified CDR positions within L3 and H3 were included for the alignment. Sequences were aligned using the Needleman–Wunsch algorithm in the EMBOSS Needle server. **(F)** Affinity and specificity of four Notch-1 Fabs isolated from Library-S master-and sub-libraries. EC_50_ and K_D_ values were determined using multi-point Fab-ELISA and bio-layer interferometry, respectively. Fab specificity was determined using single-point Fab-ELISA at 1 μM Fab concentration. Abs_450_ values are shown as a heatmap. Notch-1, Notch-2, Notch-3, Jagged-1, and Jagged-2 are indicated as N1, N2, N3, J1, and J2, respectively.

Library-S contains four-fixed CDRs (L1, L2, H1, and H2) and two-diversified CDRs (L3 and H3) on a modified Hu4D5 trastuzumab framework (Maruthachalam et al., 2017). Length diversity is included within CDRH3 and amino acid diversity is included within CDRH3 and CDRL3 (Figures 1A,B). To generate Notch-1 Fabs, we conducted solid-phase, phage display panning of Library-S against the human Notch-1 extracellular domain. Random clone picking and Sanger sequencing of 20 phagemids from the round 4 phage pool gave rise to two Fab clones (Figure 1C) N1/S/1 and N1/S/2 with CDRH3 lengths of 13 and 16 residues, respectively. Phage-ELISA indicated that both Fabs bound to Notch-1 but not to BSA or the Fc protein (Supplementary Table 2).

Since length diversity was restricted to CDRH3, we hypothesized that CDRH3 length would play a central role in Library-S binding. To develop a selection strategy for obtaining Notch-1 Fabs with different CDRH3 lengths, we split Library-S into four sub-libraries each containing a set of CDRH3 lengths, and panned them against Notch-1. After rounds 4 and 5, we calculated the number of phages eluted from target-coated wells relative to BSA-coated wells. A positive enrichment in target-specific phage number was only observed with two sub-libraries SL2 (CDRH3 range of 11–13 aa) and SL3 (CDRH3 range of 14–16 aa; Figure 1D), which contained CDRH3 lengths of Fabs (N1/S/1 and N1/S/2) isolated in the Library-S selection. Through random clone picking and Sanger sequencing we isolated Fab clones N1/SL2/1 and N1/SL3/1 from SL2 and SL3 round 4 phage selection pools, respectively (Figure 1D). N1/SL2/1 had a CDRH3 length of 13 amino acids, which was the same length as N1/S/1, and N1/SL3/1 and N1/S/2 had the same CDRH3 length of 16 residues (Figure 1E). Phage-ELISAs indicated that N1/SL2/1 and N1/SL3/1 bound to Notch-1 but not to BSA or the Fc protein (Supplementary Table 2). Random clone picking and Sanger sequencing of 20 phagemids from SL1 and SL4 round 4 selection pools gave rise to six Fab clones; however, they bound to all test and control antigens in phage-ELISA.

Next, we purified four Notch-1 Fabs and measured their affinity and specificity for Notch-1. First, we determined EC_50_ values for Fabs binding to Notch-1 using Fab-ELISA (Figure 1F; Supplementary Figure 1). The EC_50_ values for the 13 aa CDRH3 Fabs N1/S/1 and N1/SL2/1 were 3.3 nM and 49 nM, respectively. The EC_50_ of the 16 aa CDRH3 Fabs N1/S/2 and N1/SL3/1 were 7.5 nM and 12.7 nM, respectively. Second, we measured kinetics of Fab binding to Notch-1 using bio-layer interferometry. Consistent with Fab-ELISA, N1/S/1 was the highest-affinity binder (K_D_ = 0.7 nM). N1/SL2/1 bound to Notch-1 with a K_D_ value of 3.2 nM. N1/S/2 and N1/SL3/1 possessed mid-nM K_D_ values, 14 nM and 27.5 nM, respectively (Figure 1F; Supplementary Table 4). These values represent apparent K_D_ values (K_Dapp_), as the Fc-Notch proteins may dimerize through the Fc domain resulting in avidity, which may explain the lower K_D_ values observed for N1/S/1 and N1/SL2/1 compared to the EC_50_ values. In contrast, 16 aa CDRH3 Fabs had slightly higher K_Dapp_ than EC_50_ values. Third, to assess Fab specificity, we tested the binding of Fabs to Notch and Jagged receptor ectodomains using a Fab-ELISA. At 1 μM Fab concentration, N1/S/1 and N1/SL3/1 cross-reacted with Notch-3, whereas N1/SL2/1 and N1/S/2 exhibited Notch-1 specific binding (Figure 1F; Supplementary Table 3). Kinetic analysis indicated that the highest-affinity Fab N1/S/1 also bound to Notch-3 with a K_Dapp_ of 14.1 ± 0.53 nM.

### Notch-1 Fabs from Library-F

**Figure 2:**
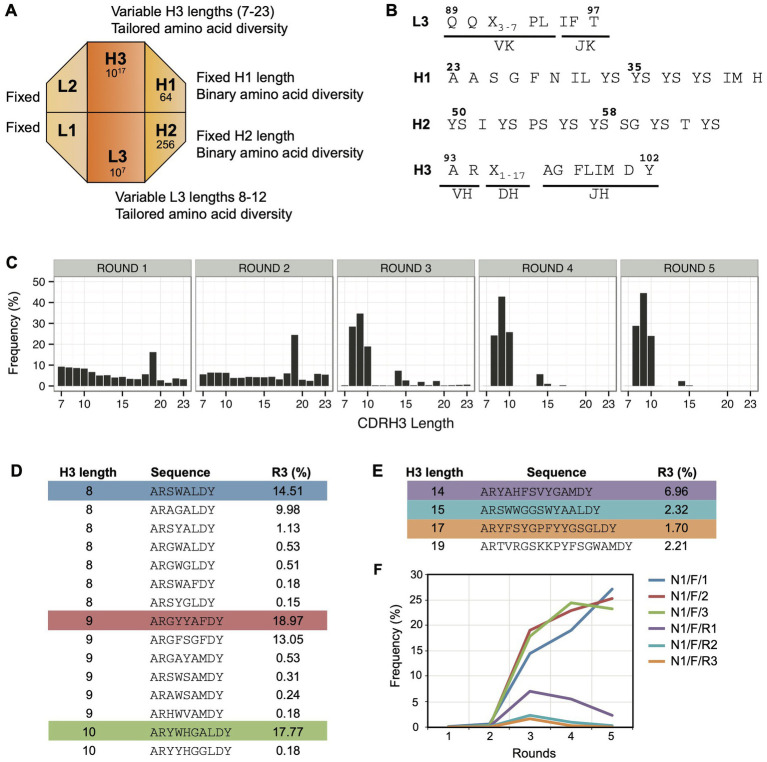
Panning Library-F against Notch-1. **(A)** Schematic representation of Library-F CDR diversity. **(B)** CDR diversity designs for L3, H1, H2, and H3. X denotes any of the following nine amino acids introduced at different proportions: Y (25%), S (20%), G (20%), A (10%), F (5%), W (5%), H (5%), P (5%), or V (5%). CDRL3 and CDRH3 lengths are varied by altering the number of X. **(C)** Changes in CDRH3 length distribution over five subsequent phage display rounds for the Notch-1 selection. The peak in rounds 1 and 2 (CDRH3 length of 19 residues) is due to the presence of anti-MBP Fab CDRH3 sequence from the template phagemid. **(D)** CDRH3 sequences >0.1% in round 3 with short loop lengths (8, 9, and 10 amino acids). **(E)** CDRH3 sequences >0.1% in round 3 with loop lengths of 14, 15, 17, and 19 amino acids. In panels **D,E**, the most abundant sequence from each CDRH3 length is highlighted. **(F)** Propagation behavior of highlighted CDRH3 sequences over five rounds of selection.

In an attempt to obtain Notch-1 Fabs with higher affinity and specificity, we used Library-F, which contains four-diversified CDRs and two-fixed CDRs on a modified trastuzumab framework (Persson et al., 2013). Length diversity is included within CDRs L3 and H3 and amino acid diversity is included within CDRs L3 and H3 and solvent-accessible positions of CDRs H1 and H2 (Figures 2A,B). We hypothesized that the additional diversity in Library-F (length diversity in CDRL3 and amino acid diversity in CDRs H1/H2) would generate higher affinity and more selective Fabs against Notch-1.

Solid-phase panning of Library-F was conducted against the Notch-1 extracellular domain. To identify Fabs with different CDRH3 lengths, we sequenced the CDRH3 region of the Fab-phage selection outputs using Ion Torrent sequencing (Rothberg et al., 2011). We monitored changes in CDRH3 length distribution over five rounds of selection (Figure 2C). We observed a prominent enrichment in three short CDRH3 lengths (8, 9, and 10 residues). Four other CDRH3 lengths (14, 15, 17, and 19 residues) were also enriched albeit at lower frequencies, ranging between 2% and 7%. We ranked round 3 CDRH3 sequences based on their relative frequencies. Nineteen sequences were present above 0.1%, and all of them had the above-mentioned CDRH3 lengths. While 15 out of 19 sequences had short CDRH3 lengths (Figure 2D), each of the other four CDRH3 lengths had one sequence above 0.1% (Figure 2E). The longest CDRH3 was 19 residues and was the anti-maltose binding protein (MBP) CDRH3 sequence used in template phagemid for library F construction (Persson et al., 2013). We analyzed the frequency of the most abundant Fabs from six selected lengths over five rounds of selection (Figure 2F). The three short CDRH3 sequences showed a significant enrichment throughout the selection process. The three larger CDRH3 sequences showed a lower enrichment in round 3, and their frequency decreased in later rounds.

**Figure 3:**
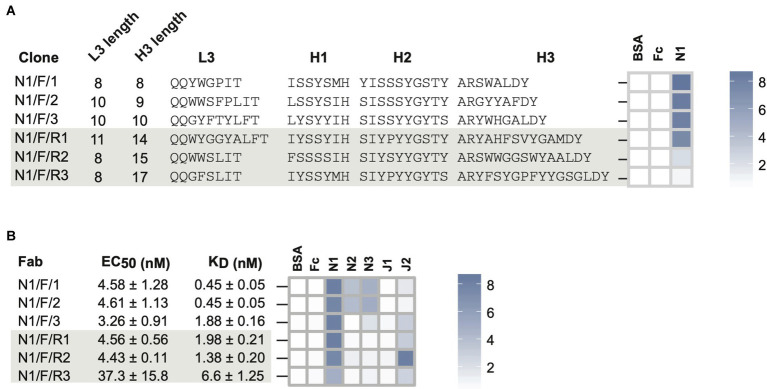
Notch-1 Fabs from Library-F. **(A)** Sequence characteristics and phage-displayed Fab binding characteristics of 3 top Fabs (N1/F/1, N1/F/2, and N1/F/3) isolated by Sanger sequencing and 3 rare Fabs (N1/F/R1, N1/F/R2, and N1/F/R3) reconstructed from L3-H1-H2-H3 NGS information. **(B)** Affinity and specificity of purified Notch-1 Fabs. Fab specificity was determined at 1 μM Fab concentration. Phage-ELISA and specificity-ELISA ABS_450_ values are shown as heatmaps. Fab nomenclature is listed as Target/Library/Clone number. Notch-1, Notch-2, Notch-3, Jagged-1, and Jagged-2 are indicated as N1, N2, N3, J1, and J2, respectively. S and F indicate library S, Library F, respectively. The clone number is listed in the third position and if they are a “rare” clone they have an “R” in front of the number.

Random clone picking and Sanger sequencing of 20 clones from rounds 3 and 4 phage pools only recovered the three most-frequent clones with short CDRH3 lengths (referred as high-frequency clones). Therefore, we used our NGS-assisted Fab reconstruction method to recover the three less-frequent clones with longer CDRH3 lengths (referred as low-frequency clones). To identify the diversified CDR sequences (L3, H1, and H2) that paired with the CDRH3 sequences of interest, we removed the intervening sequence between the CDRs using our CDR strip methodology (Barreto et al., 2019). This generated a PCR amplicon of ~200 bp that contained CDRs L3-H1-H2-H3 closer in linear sequence space. We used this strategy to sequence a CDR strip from round 3 on a 400-bp chip. As the maximum read length offered by Ion Torrent was 400 bases, the framework deletion step was necessary to reduce the amplicon length from 1,100 bases to 200 bases. Once CDR-combinations were identified, we reconstructed desired Fab clones by cloning CDR-encoding oligonucleotides into the MBP template phagemid (Persson et al., 2013) by Kunkel mutagenesis (Kunkel et al., 1987). Diversified CDR sequences of six Fab clones are shown in Figure 3A.

In phage-ELISA, all six Fabs tested positive for binding to Notch-1 (Figure 3A; Supplementary Table 2). Compared to high-frequency Fabs, ELISA signals were ~4-fold weaker for N1/F/R2 and ~ 10-fold weaker for N1/F/R3. Next, we converted six phage-Fab clones into soluble Fabs and measured their EC_50_ values against Notch-1 using Fab-ELISA (Figure 3B; Supplementary Figure 1). Except for N1/F/R3, the other five Fabs had EC_50_ values of ~4 nM. N1/F/R3 bound ~10-fold weaker than the other Fabs (EC_50_ = 37 nM). Next, we analyzed Fab-binding kinetics using bio-layer interferometry and Fab specificity using Fab-ELISA (Figure 3B; Supplementary Tables 3–4). Even though the two highest frequency Fabs, N1/F/1 and N1/F/2, exhibited sub-nM K_Dapp_ values for Notch-1, they also possessed mid-nM K_Dapp_ values for Notch-2 and low-nM values for Notch-3. N1/F/1 bound to Notch-1, Notch-2, and Notch-3 with K_Dapp_ values of 0.45 ± 0.05 nM, 48.2 ± 1.42 nM and 5.7 ± 0.23 nM, respectively. N1/F/1 affinity was 107-fold lower for Notch-2 and 13-fold lower for Notch-3. N1/F/2 bound to Notch-1, Notch-2, and Notch-3 with K_Dapp_ values of 0.45 ± 0.05 nM, 31 ± 1 nM and 4.1 ± 0.18 nM, respectively. N1/F/2 affinity was 69-fold lower for Notch-2 and 9-fold lower for Notch-3. The third highest frequency Fab N1/F/3 had a K_Dapp_ value of 1.88 nM for Notch-1 and cross-reacted with Notch-3 and Jagged-2 in Fab-ELISA. The three low-frequency Fabs with medium CDRH3 lengths possessed very similar binding profiles. They bound to Notch-1 with low-nM K_D_ values and showed weak to moderate binding with Jagged-2 in Fab-ELISA. N1/F/R1 and N1/F/R2 bound to Notch-1 with K_Dapp_ values of 1.98 nM and 1.38 nM, respectively. In agreement with phage-ELISA and Fab-ELISA, N1/F/R3 was the lowest-affinity Notch-1 binder from Library-F (K_Dapp_ = 6.6 nM). Kinetic analysis confirmed dose-dependent binding of N1/F/R2 with Jagged-2 with a K_Dapp_ value of 70.4 ± 2.3 nM.

Most notably, low-frequency Fabs recovered using the NGS approach had low-nM affinities for Notch-1 and were more specific than high-frequency Fabs isolated by Sanger sequencing. In particular, N1/F/R2 was less frequent in the round-3 phage pool (~2%), did not show enrichment in round-4 and round-5, and had weaker phage-ELISA output; however the K_Dapp_ value for purified Fab N1/F/R2 was very similar to N1/F/3 and N1/F/R1 that were more frequent in phage pools.

### Notch-1 Fabs from the Modified-F Library

**Figure 4:**
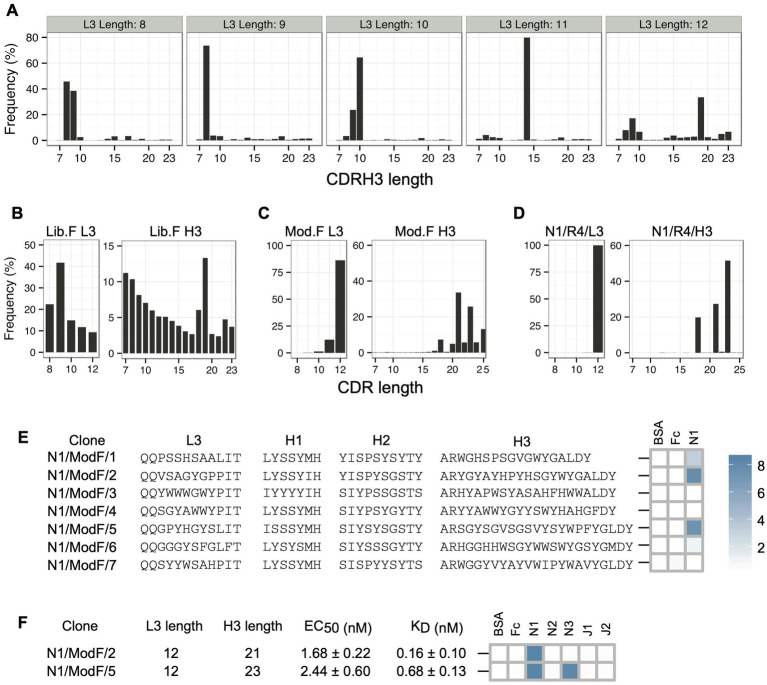
Notch-1 Fabs from the Modified-F library. **(A)** Pairing between CDRL3 lengths and CDRH3 lengths in the Notch-1 selection output (round 3) from Library-F selections. L3-H3 pairs were obtained from L3-H1-H2-H3 NGS information, and for each possible L3 length, H3 length distribution was generated. **(B)** CDRL3 and CDRH3 length distribution of the naïve Library-F. **(C)** CDRL3 and CDRH3 length distribution of the Modified-F library. **(D)** CDRL3 and CDRH3 length distribution of the Notch-1 selection output after four rounds of selection with the Modified-F library. **(E)** Diversified CDR sequences and phage-displayed Fab binding characteristics of 7 Notch-1 Fabs isolated from Modified-F selections using Sanger sequencing. **(F)** CDRL3 length, CDRH3 length, affinity, and specificity of Fabs N1/ModF/2 and N1/ModF/5. EC_50_ and K_D_ values were determined by multi-point Fab-ELISA and bio-layer interferometry, respectively. Fab specificity was determined at 1 μM Fab concentration. Phage-ELISA and specificity-ELISA ABS_450_ values are shown as heatmaps. Notch-1, Notch-2, Notch-3, Jagged-1, and Jagged-2 are indicated as N1, N2, N3, J1, and J2, respectively.

In addition to facilitating low-frequency Fab reconstruction, sequencing CDR-combinations in a selection output allows the comparison of CDRs that are paired with each other. To check the influence of CDRL3 length diversity on CDRH3 length solutions for Notch-1, we monitored the pairing between CDRL3 lengths and CDRH3 lengths in the round 3 phage selection output. We observed a preferential pairing of certain CDRL3 and CDRH3 lengths (Figure 4A). For example, CDRH3 length of eight amino acids is most-frequently paired with CDRL3 lengths of 8 and 9 amino acids. Also, the longest CDRL3 length preferred longer CDRH3 length solutions for Notch-1 recognition. In addition to slower amplification rates in *E. coli*, the poor enrichment of long CDR clones could result from the under representation of long CDR sequences in the library. To confirm this, we amplified the CDRL3 and CDRH3 regions from the naïve Library-F phage pool and sequenced them on a 100-bp Ion chip. Length analysis confirmed the presence of a bias in the library toward short sequences in both CDRs (Figure 4B). Also, we noticed high retention of template CDRL3 (~24%) and CDRH3 (~13%) sequences in the library, which has a CDRL3 length of 9 residues and CDRH3 length of 19 residues.

To isolate Notch-1 Fabs with long CDRs, we decided to modify the length bias in Library-F toward long CDRs and use the modified library for selections. The Modified-F library had the following design features: (1) CDRL3, CDRH1, and CDRH2 diversity designs were unaltered from Library-F; (2) in addition to 17 JH4-CDRH3 lengths (7–23 residues) used in Library-F, 10 JH6-CDRH3 lengths (16–25 residues) used in Library-S were included; (3) to bias the length diversity toward long CDR lengths, the amount of mutagenic oligonucleotides used in mutagenesis reactions was normalized according to their theoretical diversity; and (4) to prevent the expression of Fabs with unmutated template CDRs, the anti-MBP diversified CDR sequences (Persson et al., 2013) were replaced with TAA stop codons in the template phagemid. The Modified-F library was constructed using an M13 bacteriophage system that allows bivalent Fab display (Lee et al., 2004). Kunkel mutagenesis was used to repair stop codons in the MBP-Fab-encoding template phagemid and replace CDR positions with fixed or degenerate codons encoding the designed amino acid composition. Following mutagenesis, the library DNA was electroporated into an *E. coli* strain suitable for high-efficiency transformation and phage production. NGS analysis of the CDRL3 and CDRH3 regions of the naïve Modified-F library confirmed proper CDR diversification and the length bias toward long CDRs (Figure 4C).

We panned the Modified-F library against Notch-1 and sequenced CDRL3 and CDRH3 regions from the round 4 phage selection output on a 100-bp chip. Length analysis indicated a significant enrichment in CDRL3 sequences of 12 residues and CDRH3 sequences of 18, 21, and 23 residues (Figure 4D). Random clone picking and Sanger sequencing of 20 phagemids recovered seven unique clones from the round 4 phage pool. Three clones tested positive for binding to Notch-1 in phage-ELISA (Figure 4E and Supplementary Table 2). Each ELISA-positive clone had a different CDRH3 length and showed a high CDRH3 sequence enrichment in the round 4 phage pool (19.15% for N1/ModF/1, 25.96% for N1/ModF/2, and 43.5% for N1/ModF/5). We chose to pursue two clones that had longer CDRH3 lengths (N1/ModF/2 and N1/ModF/5). We converted phage-Fab clones into soluble Fab proteins and assayed them using Fab-ELISA (Figure 4F; Supplementary Figure 1; Supplementary Table 3). In Fab ELISA, both Fabs bound stronger to Notch-1 than all other Library-S or Library-F Fabs. The EC_50_ values for N1/ModF/2 and N1/ModF/5 were 1.7 nM and 2.4 nM, respectively. Further, N1/ModF/2 showed strict specificity for Notch-1, whereas N1/ModF/5 showed dual specificity for Notch-1 and Notch-3. During kinetic studies, N1/ModF/2 exhibited the highest affinity for Notch-1 (K_Dapp_ = 0.16 nM; Figure 4F; Supplementary Table 4). N1/ModF/2 possessed ~15-fold higher affinity than the Notch-1 specific antibody anti-NRR1 reported previously (K_D_ = 2.5 nM; Wu et al., 2010). With a sub-nM K_D_ and strict specificity, N1/ModF/2 turned out to be the best Fab clone for Notch-1. N1/ModF/5 bound to Notch-1 and Notch-3 with K_Dapp_ values of 0.68 nM and 7.1 nM, respectively.

## Discussion

Given that Library-S delivered Fabs against Jagged-1/2 and Notch-2/3 (Barreto et al., 2019), we tested whether Library-S could give rise to Fabs with high affinity and specificity against Notch-1. Library-S gave rise to a sub-nM bi-specific binder (N1/S/1) and a mid-nM mono-specific binder (N1/S/2). Next, we split Library-S into four sub-libraries each containing a set of CDRH3 lengths and panned them against Notch-1. Through sub-library screens, we expected to obtain Fabs with different CDRH3 lengths and Fabs with higher affinities compared to the master-library Fabs. Although results were contrary to our expectations, sub-library panning gave rise to a low-nM mono-specific binder (N1/SL2/1). Its corresponding master-library Fab showed ~5-fold higher affinity for Notch-1 (sub-nM K_D_), however, also had a mid-nM K_D_ for Notch-3. In going from Library-S to Library-F, we anticipated that the additional diversity in Library-F (length diversity in CDRL3 and amino acid diversity in CDRs H1/H2) would generate more potent and selective Fabs for Notch-1. Through Library-F selections, we isolated three high-frequency clones with short CDRH3 lengths by Sanger sequencing and reconstructed three low-frequency clones with medium CDRH3 lengths from NGS information. The high-frequency clones possessed sub-nM to low-nM K_D_ values for Notch-1 and cross-reacted with two other receptors. Low-frequency clones possessed low-nM to mid-nM K_D_ values for Notch-1 and cross-reacted with Jagged-2, making them bi-specific binders.

Even though CDRH3 in Library-S had higher length, amino acid, and JH segment diversities than in Library-F, Library-S selections provided only two optimal CDRH3 length solutions for Notch-1. In Library-F, the addition of length and conformational diversity to CDRs adjacent to CDRH3 increased the number of optimal CDRH3 length solutions for Notch-1. Further, CDRH3 length solutions allowed by Library-F Fabs were distinct from Library-S Fabs, indicating that length/conformational diversity in CDRs L3, H1, and H2 could also alter and determine the type of CDRH3 loop responsible for the interaction. Not only CDR lengths and amino acids, but also binding profiles of Library-F Fabs were different from Library-S Fabs, confirming previous observations that the increase in overall interface diversity fundamentally changes the nature of Fab-binding solutions rather than optimizing a common binding solution (Fellouse et al., 2007).

Since CDR length diversity in Library-F was biased toward short sequences, we designed and constructed the Modified-F library with the length bias toward long CDRs. We used the Modified-F library to isolate two Notch-1 Fabs (N1/ModF/2 and N1/ModF/5) with long CDRL3 and CDRH3 sequences. N1/ModF/2 exhibited the highest affinity for Notch-1 (K_Dapp_ = 0.16 nM) and showed strict specificity for Notch-1. N1/ModF/5 showed low-nM binding to Notch-1 and cross-reacted with Notch-3. Strikingly, binding profiles of Modified-F Fabs were very similar to Library-S Fabs. Only mono-specific Fabs (Notch-1) and bi-specific Fabs (Notch-1 and Notch-3) were isolated from both the libraries. N1/ModF/2 bound ~20-fold stronger than the best Library-S-based mono-specific Fab N1/SL2/1. By possessing the same affinity for Notch-1 (K_Dapp_ = 0.7 nM) but two-fold higher affinity for Notch-3, N1/ModF/5 performed poorly than the Library-S-based bi-specific Fab N1/S/1. While long CDR lengths contributed to the specificity of Modified-F Fabs, we speculate that the additional amino acid diversity contributed to the specificity of Library-S Fabs.

In this work, both single-framework synthetic Fab libraries (S and F) yielded sub-nM to mid-nM Notch-1 binders with different specificity profiles. However, to obtain high-affinity Notch-1 specific Fabs, screening the master library alone was not sufficient. In the case of Library-S, screening sub-libraries identified a low-nM Notch-1 specific binder. In the case of Library-F, fine-tuning the library toward long CDR lengths gave rise to a sub-nM Notch-1 specific binder. In addition to delivering Notch-1 Fabs, our work highlighted the importance of sampling a focused diversity within an antibody library. The master library only has a sparse coverage of a large diversity space, due to limitations in attainable library size. Its sub-libraries provide a denser coverage of a smaller diversity space. In contrast, affinity maturation libraries offer optimization of an existing binding solution and are constructed by random or rational diversification of an existing antibody (Marvin and Lowman, 2015).

Previous studies have shown that NGS has four main applications in antibody phage display: library quality control, analysis of selection outputs, reconstruction of low-frequency clones, and rationale design based on natural repertoires (Ravn et al., 2010; Zhang et al., 2011; Mahon et al., 2013; D'Angelo et al., 2014; Glanville et al., 2015; Yang et al., 2017; Jian et al., 2019; Valadon et al., 2019; Azevedo Reis Teixeira et al., 2021; Wang et al., 2021), which have resulted in some FDA approved antibodies (Alfaleh et al., 2020). In this work, we used NGS information for identifying and reconstructing low-frequency clones with less-frequent CDRH3 lengths, for studying the pairing between CDRL3 and CDRH3 lengths in the selection output, and for fine-tuning the library length diversity. These advances in the use of NGS information gave rise to Fab clones with better binding properties. We used CDRH3 length as a parameter for designing and screening libraries and for isolating Fabs from selection outputs. This approach proved to be successful for obtaining Fabs with different and superior binding profiles that may prove useful in clinical applications.

The isolation of antibodies with new epitopes and binding profiles against Notch pathway members are of interest for designing new antibodies for clinical studies. Antibodies targeting the Notch pathway first entered clinical trials in 2010 and had limited success. One reason for their failure is likely due to the complex nature of this signaling pathway, which can be both pro-and anti-tumor depending on the tumor microenvironment (Zhou et al., 2022). Antibodies targeting Notch1 (brontictuzumab; NCT01703572, NCT01778439, NCT02662608, NCT03031691) or Notch2/3 (tarextumab; NCT01277146, NCT01859741; NCT01647828) receptors have failed in phase I and phase II clinical trials due to lack of efficacy (Ferrarotto et al., 2018; Hu et al., 2019). Clinical trials against Notch receptor ligands DLL3 (rovalpituzumab tesirine; Uprety et al., 2021) and DLL4 (enoticumab (Chiorean et al., 2015) and demcizumab (Coleman et al., 2020)) also showed poor efficacy. Current Notch pathway antibodies in clinical trials, are bi-specific or tri-specific. These antibodies include bispecific DLL4/VEGFA targeting antibodies navicixizumab (NCT05043402) and dilpacimab (NCT01946074), bi-specific targeting DLL3/CD3ε antibody tarlatamab (NCT05060016, NCT05361395, NCT04702737, and NCT04885998) and the tri-specific targeting DLL3/CD3ε/albumin antibody HPN328 (NCT04471727). Multi-specific antibodies should improve their tumor targeting and the targeting of T-cells to tumors.

In summary, we used synthetic antibody technology for generating selective Fabs against the extracellular domain of Notch-1. Twelve Fabs with 10 different CDRH3 lengths were identified from single-framework synthetic Fab libraries using phage display. Upon testing, Fabs showed high affinity for Notch receptors (sub-nM to mid-nM K_Dapp_ values) and exhibited different binding profiles (mono-or bi-or tri-specific) toward Notch/Jagged receptors. Most likely, these Fabs recognize different epitopes on Notch-1 and could be used for modulating the Notch signaling pathway using different mechanisms of action. Two Fabs exhibited strict specificity for Notch-1 with low nanomolar K_Dapp_ values. In contrast to gene knockout approaches, γ-secretase inhibitors, and pan-Notch antibodies (Espinoza and Miele, 2013; Takebe et al., 2014), our mono-specific Fabs may permit a more precise control of Notch-1 inhibition. Over the course of Fab generation, we also showed that implementing NGS approaches, screening focused diversity libraries, and fine-tuning the library diversity can improve the success rate of single-framework synthetic Fab libraries. These findings have valuable implications for antibody library design, antibody phage display, and combinatorial antibody engineering.

## Materials and methods

### Construction of single-framework synthetic Fab libraries

Library-S is previously described and contains four-fixed CDRs (L1, L2, H1, and H2) and two-diversified CDRs (L3 and H3) on a modified trastuzumab framework (Maruthachalam et al., 2017).

The modified library (Modified-F) was constructed as follows. The template phagemid used for constructing the Modified-F Fab library was derived from the phagemid encoding the anti-maltose binding protein (MBP) Fab from Library-F (Persson et al., 2013). Kunkel mutagenesis (Kunkel et al., 1987) was used to replace four CDRs (L3, H1, H2, and H3) in the MBP phagemid with TAA stop codons. The resulting phagemid was sequence verified and used as the template phagemid. The Modified-F library was constructed and stored using previously established protocols (Fellouse and Sidhu, 2006; Rajan and Sidhu, 2012). Kunkel mutagenesis was used to simultaneously diversify four CDR regions (L3, H1, H2, and H3) within the template phagemid. CDRs L3, H1, and H2 were diversified using Library-F mutagenic oligonucleotides. CDRH3 was diversified using 17 J_H_4-CDRH3 Library-F mutagenic oligonucleotides and 10 J_H_6-CDRH3 Library-S mutagenic oligonucleotides (Oligonucleotide sequences given in Supplementary Table 1). Eight different mutagenesis reactions were required for constructing the Modified-F library, each reaction representing a set of CDRH3 lengths. DNA from the mutagenesis reaction was purified using the PCR cleanup kit and 10 μg of purified DNA was electroporated into M13KO7-infected SR320 *E. coli* cells for phage production. Phages were purified from the culture supernatant using PEG/NaCl precipitation, resuspended in PBS and stored at-80°C in the presence of protease inhibitors (2%) and sterile glycerol (25%). Phages from eight sub-libraries were rescued separately and an equal number of phages from each sub-library (~5 × 10^13^ PFU) was mixed together to create the Modified-F library.

### Phage display selections

The recombinant Fc-tag-fused human Notch-1 extracellular domain was purchased from R&D Systems. Solid-phase panning of phage-displayed Fab libraries (S, F, and modified-F) was conducted according to previously described protocols (Fellouse and Sidhu, 2006; Rajan and Sidhu, 2012). Briefly, phages from the frozen master library were precipitated, deselected for binding to the Fc protein and cycled through rounds of binding selection with Notch-1 coated on 96-well MaxiSorp plates and amplification of Notch-1-bound phage in XL1-Blue *E. coli* cells. After four rounds of selections, phage clones were plated as individual colonies for isolation, sequencing, and manipulation of phagemid DNA.

### Ion Torrent sequencing and data analysis

Ion Torrent sequencing of one diversified CDR (L3 or H3) was accomplished in three steps: PCR amplification of CDR, emulsion PCR on Ion sphere particles (ISPs), and sequencing enriched ISPs on an Ion semiconductor chip. To PCR amplify CDRs from phage pools, we designed primers that hybridize to the fixed framework regions of the phagemid that flank the CDR region. Primers contain barcodes for multiplexing purposes and adapter sequences to facilitate emulsion PCR. We PCR-amplified the CDR of interest from phage samples, checked the purity, concentration, and length of PCR products using a 2,100 bio-analyzer (Agilent Technologies), prepared the template for emulsion PCR by pooling multiple PCR products, performed emulsion amplification of the amplicon library on the Ion OneTouch 2 instrument (Life Technologies) using the Ion OneTouch template kit, loaded the enriched ISPs into an Ion 314 Semiconductor chip, and sequenced the loaded ISPs on the V2 Ion Personal Genome Machine (Thermo Scientific) using the Ion PGM supplies kit.

Ion Torrent sequencing of the L3-H1-H2-H3 CDR strip was accomplished in six steps. (1) ssDNA was extracted from amplified phage selection outputs (10^13^ PFU) using the Spin M13 kit (Qiagen). (2) 500 ng of ssDNA was subjected to Kunkel mutagenesis for deleting the framework regions between four diversified CDRs. In the mutagenesis reaction, three oligonucleotides, L3-H1 Seq, H1-H2 Seq, and H2-H3 Seq, were used to link the L3-H1-H2-H3 regions together. Phosphorylation of oligonucleotides, annealing of oligonucleotides to the ssDNA template, and *in vitro* synthesis of CCC-dsDNA were carried out as described previously (Tonikian et al., 2007; Nelson and Sidhu, 2012). (3) DNA from the mutagenesis reaction was run on an agarose gel and the right-sized product (CCC-dsDNA) was excised and purified using a gel extraction kit. (4) The L3-H1-H2-H3 CDR strip was PCR amplified from 50 ng of purified CCC-dsDNA using barcoded L3-Fwd and H3-Rev primers. (5) PCR amplicons were purified, quantified, multiplexed, and subjected to emulsion PCR using the Ion PGM Template OT2 400 kit. (6) Enriched ISPs were loaded on an Ion 314 Chip and sequenced using the Ion PGM Sequencing 400 kit.

We built a custom workflow for NGS data processing and analysis (Barreto et al., 2019). Sequences were base called and separated by the barcode on the Ion PGM Torrent Server and exported in FASTQ format. Sequences were imported into the Galaxy server (Blankenberg et al., 2010; Goecks et al., 2010), where they were trimmed based on quality score (>17), converted to FASTA, and then run on a custom R script (R Core Team, 2013) to parse the CDR, translate, and perform sequence counts. CDR sequences were processed using the Biostrings package (Pagès et al., 2016) and length distribution plots were generated using the ggplot2 package (Wickham, 2009).

### Reconstruction of rare Notch-1 Fabs

To reconstruct rare Notch-1 Fab clones from the CDR strip sequencing information, we cloned desired CDR combinations into the MBP-encoding phagemid by Kunkel mutagenesis (Kunkel et al., 1987). The MBP phagemid CDRs were replaced with *NotI* sites and used as a template to reconstruct Fab clones. Oligonucleotides were designed to encode for a desired CDR sequence and to hybridize to either side of the CDR. Four oligonucleotides were used in the Kunkel mutagenesis reaction, one for each diversified CDR. Phosphorylation of oligonucleotides, annealing of oligonucleotides to the uracil-inserted ssDNA template, and *in vitro* synthesis of CCC-dsDNA were carried out as described previously (Tonikian et al., 2007; Nelson and Sidhu, 2012). Following mutagenesis, the reaction was transformed into *dut*^+^/*ung*^+^ *E. coli* to eliminate the wild-type template strand. Positive Fab clones (N1/F/R1, N1/F/R2, and N1/F/R3) were screened by *NotI* restriction digestion analysis.

### Sub-cloning, expression, and purification of Notch-1 Fabs

Notch-1 Fabs were sub-cloned from the phagemid vector into a modified pCW-LIC Fab expression vector using standard molecular biology procedures. Briefly, Fab sequences were amplified from phagemids by PCR, and ligated into the *SacI*/*XhoI*-digested pCW-LIC vector using Gibson assembly (Gibson et al., 2009). Gibson assembly reactions were electroporated into BL21 *E. coli* cells, and three colonies from each reaction were screened for Fab expression using bio-layer interferometry in 96-well format. Briefly, single colonies were transferred to 1 ml of Overnight Express Terrific Broth (TB) auto-induction medium (EMD Millipore) supplemented with 100 μg/ml carbenicillin in 96-well deep-well boxes and incubated for 18 h at 25°C and 200 rpm. Cells were pelleted by centrifugation and lysed with 200 μl of B-PER bacterial protein extraction reagent (Pierce). Cells were centrifuged again, and 50 μl of the clarified supernatant was transferred to 384-well plates. Fab expression was detected using anti-Fab C_H_1 biosensors and anti-HIS biosensors in the ForteBio Octet RED384 system (Pall Corporation) according to the manufacturer’s instructions. Positive clones were transferred into 30 ml of TB auto-induction medium supplemented with 100 μg/ml carbenicillin and incubated for 18 h at 25°C with shaking at 200 rpm. Cells were pelleted by centrifugation, and lysed in protein-L binding buffer (20 mM Na_2_HPO_4,_ 0.15 M NaCl, pH 8) containing 1:100 dilution of protease inhibitor cocktail (Sigma) using a cell disruptor (Constant Systems) Clarified supernatant was incubated with 200 μl of Protein-L resin (GenScript) for 1 h at 4°C. The Protein-L resin was collected by centrifugation and washed 5X with Protein-L binding buffer. Fabs were eluted with IgG elution buffer (Thermo-Scientific) and neutralized with 1 M Tris–HCl (pH 9). Eluted Fabs were dialyzed against PBS and stored at −20°C. Fab purity was verified using a 2,100 bio-analyzer and Fab concentration was determined by UV–visible spectrometry.

### Enzyme-linked immunosorbent assays

Phage-ELISA was performed to check the binding of phage-displayed Fabs to immobilized target and control proteins. The Fab-encoding phagemid was electroporated into M13KO7-infected electro-competent SR320 *E. coli* cells for phage production. The cells were rescued with pre-warmed SOC media and incubated for 30 min at 37°C. The culture was transferred to 30 ml of 2YT media supplemented with 100 μg/ml carbenicillin and 25 μg/ml kanamycin and incubated overnight at 37°C with shaking at 200 rpm. Phages were precipitated from the culture supernatant using 6 ml of ice-cold PEG/NaCl solution, resuspended in PBT buffer (PBS containing 0.5% BSA and 0.05% Tween), and quantified using UV spectrometry.

To conduct phage-ELISA, Notch-1 and control proteins were immobilized at 5 μg/ml on MaxiSorp plates (Nunc) by overnight incubation at 4°C. The wells were subsequently blocked with PB buffer (PBS containing 0.5% BSA) for 90 min at RT before washing four times with PT buffer (PBS containing 0.05% Tween). Wells were then exposed to PBT-diluted phage solution (10^12^ PFU/ml) for 30 min, washed 8X with PT buffer, and then incubated with a 1:3,000 dilution of HRP-conjugated anti-M13 antibody (GE healthcare) for 30 min at RT. Plates were washed again 6X with PT buffer and 2X with PBS. Wells were developed with 3,3′,5,5′-tetramethylbenzidine (TMB) substrate for 5 min and quenched with an equal volume of 1 M H_3_PO_4_. The plates were read at 450 nm using a SpectraMax 340PC plate reader (Molecular devices). Phage-ELISA values are provided in Supplementary Table 2.

Fab-ELISA was performed to check the binding of purified Fabs to immobilized target proteins. Proteins were immobilized at 5 μg/ml on MaxiSorp plates by overnight incubation at 4°C. Wells were subsequently blocked with PB buffer for 90 min at RT before washing 4X with PT buffer. Wells were then exposed to 100 μl of Fab solution diluted in PT for 30 min, washed 10X with PT buffer, and then incubated with a 1:3,000 dilution of HRP-conjugated anti-HIS antibody (Rockland Biosciences) for 30 min at RT. Plates were washed again X6 with PT buffer and X2 with PBS. Wells were developed with TMB substrate for 5 min and quenched with an equal volume of 1 M H_3_PO_4_. Plates were read at 450 nm using a SpectraMax 340PC plate reader (Molecular devices). Single-point Fab-ELISA was used to assess Fab specificity at 1 μM Fab concentration, and multi-point Fab-ELISA was used to calculate the EC_50_ for Fab binding to the immobilized target. In multi-point Fab ELISA, ABS_450_ values were obtained for a range of Fab concentrations (0.01 nM–1 μM) and the EC_50_ was calculated by fitting the data to the one-site specific-binding equation in Prism (Graphpad). Single-point Fab-ELISA values are provided in Supplementary Table 3 and multi-point Fab ELISA fits are provided in Supplementary Figure 1.

### Bio-layer interferometry

The ForteBio Octet RED384 system (Pall Corporation) was used to measure the binding kinetics between purified Notch-1 Fabs and target proteins. Fabs were immobilized on amine-reactive generation-2 biosensors or anti-Fab CH1 biosensors according to the manufacturer’s instructions. Immobilized Fabs were exposed to increasing concentrations of target proteins, and association and dissociation rates were measured by the shift in wavelength (nm). All reactions were performed at 25°C in PBS. For each sensor-immobilized Fab, at least three different target protein concentrations were used, and K_D_ (equilibrium dissociation constant) was obtained by globally fitting the data to a 1:1 binding model. Data were collected with Octet Data Acquisition version 7.1.0.87 (ForteBio) and analyzed using Octet Data Analysis version 7.1 (ForteBio). The list of K_on_, K_off_ and K_Dapp_ values is provided in Supplementary Table 4.

## Data availability statement

The raw data supporting the conclusions of this article will be made available by the authors, without undue reservation.

## Author contributions

BM and CG conceived the idea. CG supervised the entire study. BM conducted the experiments and analyzed the results with KB, DH, and AK. AK and KB supervised bioinformatics experiments. BM and CG wrote the manuscript with additions from KB. All authors contributed to the article and approved the submitted version.

## Funding

Funding for this work came from Western Economic Diversification Canada 12939, Natural Sciences and Engineering Research Council, grant number 027-25634, and Royal University Hospital Foundation – Nutrien Clinical Research Chair. BM thanks the Department of Biochemistry, University of Saskatchewan and the Government of Saskatchewan for Graduate Scholarships and Awards.

## Conflict of interest

The authors declare that the research was conducted in the absence of any commercial or financial relationships that could be construed as a potential conflict of interest.

## Publisher’s note

All claims expressed in this article are solely those of the authors and do not necessarily represent those of their affiliated organizations, or those of the publisher, the editors and the reviewers. Any product that may be evaluated in this article, or claim that may be made by its manufacturer, is not guaranteed or endorsed by the publisher.
