# Supplementary material for: Generation of synthetic antibody fragments with optimal complementarity determining region lengths for Notch-1 recognition
Source: Front Microbiol. 2022 Aug 3;13:931307. doi: 10.3389/fmicb.2022.931307 (PMC9381698; doi:10.3389/fmicb.2022.931307)
Supplement: Supplementary file 1 [file Data_Sheet_1.docx]

**Supplementary Table 1: List of oligonucleotides used for diversifying CDRs in the Modified-F library**

**F-L3-08:** GCAACTTATTACTGTCAGCAA**XXXST**ACGTTCGGACAGGG

**F-L3-09:** GCAACTTATTACTGTCAGCAA**XXXXST**ACGTTCGGACAGGG

**F-L3-10:** GCAACTTATTACTGTCAGCAA**XXXXXST**ACGTTCGGACAGGG

**F-L3-11:** GCAACTTATTACTGTCAGCAA**XXXXXXST**ACGTTCGGACAGGG

**F-L3-12:** GCAACTTATTACTGTCAGCAA**XXXXXXXST**ACGTTCGGACAGGG

**F-H1-13:** GCAGCTTCTGGCTTCAAC**KWWWWQ**CACTGGGTGCGTCAG

**F-H2-10:** GCCTGGAATGGGTTGCA**W**ATT**WNWWRW**ACT**W**TATGCCGATAGCGTC

**F-H3-JH4-07:** CGTCTATTATTGTGCTCGC**XEF**TTCGACTACTGGGGTCAAG

**F-H3-JH4-08:** CGTCTATTATTGTGCTCGC**XXEF**TTCGACTACTGGGGTCAAG

**F-H3-JH4-09:** CGTCTATTATTGTGCTCGC**XXXEF**TTCGACTACTGGGGTCAAG

**F-H3-JH4-10:** CGTCTATTATTGTGCTCGC**XXXXEF**TTCGACTACTGGGGTCAAG

**F-H3-JH4-11:** CGTCTATTATTGTGCTCGC**XXXXXEF**TTCGACTACTGGGGTCAAG

**F-H3-JH4-12:** CGTCTATTATTGTGCTCGC**XXXXXXEF**TTCGACTACTGGGGTCAAG

**F-H3-JH4-13:** CGTCTATTATTGTGCTCGC**XXXXXXXEF**TTCGACTACTGGGGTCAAG

**F-H3-JH4-14:** CGTCTATTATTGTGCTCGC**XXXXXXXXEF**TTCGACTACTGGGGTCAAG

**F-H3-JH4-15:** CGTCTATTATTGTGCTCGC**XXXXXXXXXEF**TTCGACTACTGGGGTCAAG

**F-H3-JH4-16:** CGTCTATTATTGTGCTCGC**XXXXXXXXXXEF**TTCGACTACTGGGGTCAAG

**F-H3-JH4-17:** CGTCTATTATTGTGCTCGC**XXXXXXXXXXXEF**TTCGACTACTGGGGTCAAG

**F-H3-JH4-18:** CGTCTATTATTGTGCTCGC**XXXXXXXXXXXXEF**TTCGACTACTGGGGTCAAG

**F-H3-JH4-19:** CGTCTATTATTGTGCTCGC**XXXXXXXXXXXXXEF**TTCGACTACTGGGGTCAAG

**F-H3-JH4-20:** CGTCTATTATTGTGCTCGC**XXXXXXXXXXXXXXEF**TTCGACTACTGGGGTCAAG

**F-H3-JH4-21:** CGTCTATTATTGTGCTCGC**XXXXXXXXXXXXXXXEF**TTCGACTACTGGGGTCAAG

**F-H3-JH4-22:** CGTCTATTATTGTGCTCGC**XXXXXXXXXXXXXXXXEF**TTCGACTACTGGGGTCAAG

**F-H3-JH4-23:** CGTCTATTATTGTGCTCGC**XXXXXXXXXXXXXXXXXEF**TTCGACTACTGGGGTCAAG

**S-H3-JH6-16:** ccgtctattattgtgctcgc**XXXXXXX**tactactactactttgactactggggtcaaggaaccct

**S-H3-JH6-17:** ccgtctattattgtgctcgc**XXXXXXXX**tactactac**JO**gac**U**tggggtcaaggaaccct

**S-H3-JH6-18:** ccgtctattattgtgctcgc**XXXXXXXXX**tactactac**JO**gac**U**tggggtcaaggaaccct

**S-H3-JH6-19:** ccgtctattattgtgctcgc**XXXXXXXXX**tactactactac**JO**gac**U**tggggtcaaggaaccct

**S-H3-JH6-20:** ccgtctattattgtgctcgc**XXXXXXXXXX**tactactactac**JO**gacgtttggggtcaaggaaccct

**S-H3-JH6-21:** ccgtctattattgtgctcgc**XXXXXXXXXXX**tactactactac**JO**gacgtttggggtcaaggaaccct

**S-H3-JH6-22:** ccgtctattattgtgctcgc**XXXXXXXXXXXX**tactactactac**JO**gacgtttggggtcaaggaaccct

**S-H3-JH6-23:** ccgtctattattgtgctcgc**XXXXXXXXXXXXX**tactactactacgga**O**gacgtttggggtcaaggaaccct

**S-H3-JH6-24:** ccgtctattattgtgctcgc**XXXXXXXXXXXXXX**tactactactacgga**O**gacgtttggggtcaaggaaccct

**S-H3-JH6-25:** ccgtctattattgtgctcgc**XXXXXXXXXXXXXXX**tactactactacggaatggacgtttggggtcaaggaaccct

34 mutagenic oligonucleotides were used for diversifying CDRs in the modified-F library. Diversified positions within mutagenic oligonucleotides are colored in red. Codon X denotes any of the following nine amino acids introduced at different proportions: Y (25%), S (20%), G (20%), A (10%), F (5%), W (5%), H (5%), P (5%) or V (5%). Codon S encodes for two amino acids P or L at 50% each. Codon T encodes for two amino acids I or F at 50% each. Codon K encodes for two amino acids I or L at 50% each. Codon W encodes for two amino acids Y or S at 50% each. Codon Q encodes for two amino acids I or M at 50% each. Codon N encodes for two amino acids P or S at 50% each. Codon R encodes for two amino acids G or S at 50% each. Codon E encodes for two amino acids A or G at 50% each. Codon F encodes for four amino acids F, L, I or M at 25% each. Codon J encodes for two amino acids G or Y at 50% each. Codon O encodes for two amino acids M or F at 50% each. Codon U encodes for two amino acids V or Y at 50% each.

**Supplementary Table 2: Phage-ELISA ABS_450_ Values**

| **Phage** | **BSA** | **FC** | **Notch-1** |
| --- | --- | --- | --- |
| N1/S/1 | 0.11 | 0.10 | 8.38 |
| N1/S/2 | 0.10 | 0.12 | 8.22 |
| N1/SL2/1 | 0.11 | 0.12 | 9.06 |
| N1/SL3/1 | 0.11 | 0.11 | 8.18 |
| N1/F/1 | 0.12 | 0.17 | 8.89 |
| N1/F/2 | 0.11 | 0.12 | 8.49 |
| N1/F/3 | 0.13 | 0.12 | 8.12 |
| N1/F/R1 | 0.16 | 0.12 | 7.44 |
| N1/F/R2 | 0.16 | 0.13 | 2.09 |
| N1/F/R3 | 0.12 | 0.16 | 0.85 |
| N1/ModF/1 | 0.12 | 0.14 | 3.31 |
| N1/ModF/2 | 0.11 | 0.12 | 8.08 |
| N1/ModF/3 | 0.25 | 0.27 | 0.17 |
| N1/ModF/4 | 0.13 | 0.14 | 0.11 |
| N1/ModF/5 | 0.11 | 0.12 | 7.64 |
| N1/ModF/6 | 0.12 | 0.13 | 1.01 |
| N1/ModF/7 | 0.20 | 0.61 | 0.18 |

Phage-ELISA was used to test binding of phage-displayed Fab to Notch-1 at 10^11^ PFU/well.

See Methods (Enzyme-Linked Immunosorbent Assays) for more details.

**Supplementary Table 3: Single-Point Fab-ELISA ABS_450_ Values**

| **Fab** | **BSA** | **FC** | **Notch-1** | **Notch-2** | **Notch-3** | **Jagged-1** | **Jagged-2** |
| --- | --- | --- | --- | --- | --- | --- | --- |
| N1/S/1 | 0.11 | 0.12 | 4.46 | 0.30 | 3.81 | 0.20 | 0.23 |
| N1/S/2 | 0.11 | 0.13 | 7.69 | 0.21 | 0.33 | 0.15 | 0.17 |
| N1/SL2/1 | 0.11 | 0.11 | 2.96 | 0.13 | 0.33 | 0.11 | 0.18 |
| N1/SL3/1 | 0.15 | 0.21 | 6.40 | 0.41 | 1.65 | 0.56 | 0.81 |
| N1/F/1 | 0.13 | 0.29 | 8.62 | 3.83 | 4.92 | 0.34 | 1.52 |
| N1/F/2 | 0.14 | 0.34 | 8.07 | 4.22 | 5.24 | 0.27 | 0.87 |
| N1/F/3 | 0.20 | 0.46 | 8.56 | 0.32 | 1.93 | 0.66 | 3.10 |
| N1/F/R1 | 0.12 | 0.23 | 8.70 | 0.48 | 0.48 | 0.44 | 3.17 |
| N1/F/R2 | 0.23 | 0.40 | 7.99 | 1.21 | 0.97 | 0.94 | 8.65 |
| N1/F/R3 | 0.21 | 0.51 | 5.12 | 0.59 | 0.98 | 0.54 | 2.85 |
| N1/ModF/2 | 0.12 | 0.18 | 9.05 | 0.12 | 0.23 | 0.14 | 0.25 |
| N1/ModF/5 | 0.11 | 0.14 | 9.29 | 0.46 | 8.62 | 0.12 | 0.25 |

Single-point Fab-ELISA was used to assess Fab specificity at 1 μM Fab concentration.

See Methods (Enzyme-Linked Immunosorbent Assays) for more details.

**Supplementary Table 4: K_on_ and K_off_ values for K_Dapp_ values reported in the manuscript**

| **Fab** | **Target** | **K_D_ (M)** | **K_D_ error** | **K_on_ (1/Ms)** | **K_on_ error** | **K_off_ (1/s)** | **K_off_ error** |
| --- | --- | --- | --- | --- | --- | --- | --- |
| N1/S/1 | Notch-1 | 7.02E-10 | 3.58E-10 | 4.51E+04 | 7.42E+02 | 3.17E-05 | 1.61E-05 |
| N1/S/1 | Notch-3 | 1.41E-08 | 5.26E-10 | 3.86E+04 | 4.27E+02 | 5.44E-04 | 2.00E-05 |
| N1/S/2 | Notch-1 | 1.40E-08 | 1.36E-09 | 3.23E+04 | 5.04E+02 | 4.92E-04 | 4.16E-05 |
| N1/SL2/1 | Notch-1 | 3.16E-09 | 6.18E-10 | 2.92E+04 | 4.23E+02 | 8.96E-05 | 1.67E-05 |
| N1/SL3/1 | Notch-1 | 2.75E-08 | 9.29E-10 | 4.32E+04 | 5.15E+02 | 1.15E-03 | 3.38E-05 |
| N1/F/1 | Notch-1 | 4.45E-10 | 5.22E-11 | 2.96E+05 | 3.45E+03 | 1.18E-04 | 1.27E-05 |
| N1/F/1 | Notch-2 | 4.82E-08 | 1.42E-09 | 1.65E+05 | 3.71E+03 | 7.49E-03 | 1.33E-04 |
| N1/F/1 | Notch-3 | 5.70E-09 | 2.27E-10 | 2.23E+05 | 2.98E+03 | 1.14E-03 | 3.98E-05 |
| N1/F/2 | Notch-1 | 4.45E-10 | 4.77E-11 | 3.22E+05 | 3.46E+03 | 1.23E-04 | 1.27E-05 |
| N1/F/2 | Notch-2 | 3.10E-08 | 9.98E-10 | 2.00E+05 | 4.69E+03 | 5.94E-03 | 1.25E-04 |
| N1/F/2 | Notch-3 | 4.10E-09 | 1.75E-10 | 2.21E+05 | 2.39E+03 | 8.59E-04 | 3.15E-05 |
| N1/F/3 | Notch-1 | 1.88E-09 | 1.60E-10 | 1.22E+05 | 1.98E+03 | 2.18E-04 | 1.88E-05 |
| N1/F/R1 | Notch-1 | 1.98E-09 | 2.07E-10 | 7.64E+04 | 1.17E+03 | 1.54E-04 | 1.47E-05 |
| N1/F/R2 | Notch-1 | 1.38E-09 | 1.99E-10 | 5.44E+04 | 5.10E+02 | 6.91E-05 | 1.05E-05 |
| N1/F/R2 | Jagged-2 | 7.04E-08 | 2.28E-09 | 3.13E+05 | 8.33E+03 | 2.26E-02 | 3.87E-04 |
| N1/F/R3 | Notch-1 | 6.61E-09 | 1.25E-09 | 8.53E+04 | 1.24E+03 | 4.84E-04 | 8.76E-05 |
| N1/ModF/2 | Notch-1 | 1.58E-10 | 1.01E-10 | 1.58E+05 | 2.89E+03 | 1.91E-05 | 1.46E-05 |
| N1/ModF/5 | Notch-1 | 6.78E-10 | 1.29E-10 | 1.24E+05 | 1.88E+03 | 8.15E-05 | 1.55E-05 |
| N1/ModF/5 | Notch-3 | 7.07E-09 | 4.51E-10 | 7.09E+04 | 8.68E+02 | 5.01E-04 | 3.11E-05 |


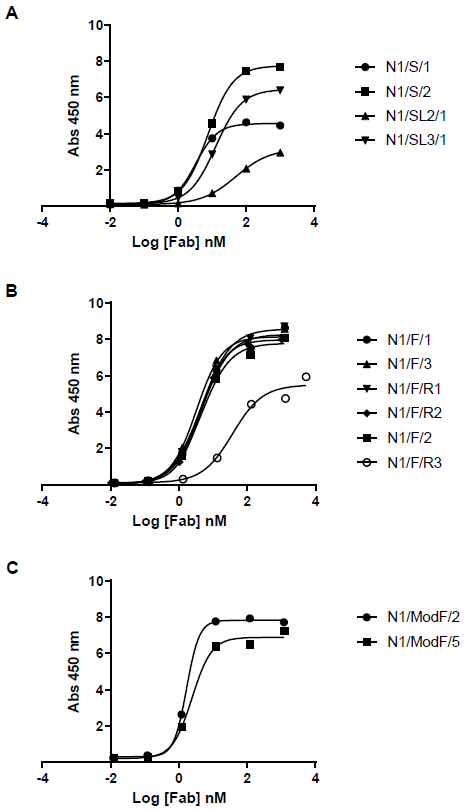


**Supplementary Figure 1:** Analysis of Notch-1 Fabs binding to immobilized Notch-1 by multi-point Fab-ELISA. **A:** Library-S Fabs; **B:** Library-F Fabs; and **C:** Fabs from modified library-F. EC_50_ values were calculated by fitting the data to the one-site specific-binding equation. EC_50_ values are shown in Figures 1, 3 and 4 for S, F and Modified-F Fabs, respectively.
